# HTR2B as a novel biomarker of chronic obstructive pulmonary disease with lung squamous cell carcinoma

**DOI:** 10.1038/s41598-024-63896-x

**Published:** 2024-06-08

**Authors:** Yue Li, Yu Wang, Ruhao Wu, Pengfei Li, Zhe Cheng

**Affiliations:** Department of Pulmonary and Critical Care Medicine, The First Affiliated Hospital of Zhengzhou University, Zhengzhou, 450052 Henan China

**Keywords:** COPD, LUSC, Machine learning, EMT, Biomarker, Cancer, Cell biology, Computational biology and bioinformatics, Genetics, Biomarkers, Oncology

## Abstract

Chronic obstructive pulmonary disease (COPD) is often associated with lung squamous cell carcinoma (LUSC), which has the same etiology (smoking, inflammation, oxidative stress, microenvironmental changes, and genetics). Smoking, inflammation, and airway remodeling are the most important and classical mechanisms of COPD comorbidity in LUSC patients. Cancer can occur during repeated airway damage and repair (airway remodeling). Changes in the inflammatory and immune microenvironments, which can cause malignant transformation of some cells, are currently being revealed in both LUSC and COPD patients. We obtained the GSE76925 dataset from the Gene Expression Omnibus database. Screening for possible COPD biomarkers was performed using the LASSO regression model and a random forest classifier. The compositional patterns of the immune cell fraction in COPD patients were determined using CIBERSORT. HTR2B expression was analyzed using validation datasets (GSE47460, GSE106986, and GSE1650). HTR2B expression in COPD cell models was determined via real-time quantitative PCR. Epithelial–mesenchymal transition (EMT) marker expression levels were determined after knocking down or overexpressing HTR2B. HTR2B function and mechanism in LUSC were analyzed with the Kaplan‒Meier plotter database. HTR2B expression was inhibited to detect changes in LUSC cell proliferation. A total of 1082 differentially expressed genes (DEGs) were identified in the GSE76925 dataset (371 genes were significantly upregulated, and 711 genes were significantly downregulated). Kyoto Encyclopedia of Genes and Genomes pathway enrichment analysis indicated that the DEGs were mainly enriched in the p53 signaling and β-alanine metabolism pathways. Gene Ontology enrichment analysis indicated that the DEGs were largely related to transcription initiation from the RNA polymerase I promoter and to the regulation of mononuclear cell proliferation. The LASSO regression model and random forest classifier results revealed that HTR2B, DPYS, FRY, and CD19 were key COPD genes. Immune cell infiltration analysis indicated that these genes were closely associated with immune cells. Analysis of the validation sets suggested that HTR2B was upregulated in COPD patients. HTR2B was significantly upregulated in COPD cell models, and its upregulation was associated with increased EMT marker expression. Compared with that in bronchial epithelial cells, HTR2B expression was upregulated in LUSC cells, and inhibiting HTR2B expression led to the inhibition of LUSC cell proliferation. In conclusions, HTR2B might be a new biomarker and therapeutic target in COPD patients with LUSC.

## Introduction

Chronic obstructive pulmonary disease (COPD) is an inflammatory lung disease that is mainly caused by chronic smoking and is characterized by irreversible airflow restriction^1^. COPD is the most disabling and fatal noncommunicable disease and has become one of the top three causes of mortality, with approximately 3 million people dying from COPD each year worldwide^2^. The current gold standard for diagnosing COPD is testing pulmonary function. However, COPD may be latent in heavy smokers who may even have normal lung function; by the time symptoms, such as difficulty breathing, develop, lung function is often reduced to 50%^3^. Therefore, it is highly important to search for COPD biomarkers.

Lung cancer is a common malignant tumor, and non-small cell lung cancer (NSCLC) accounts for > 80% of cases^4^. The incidence of lung cancer is the highest among all malignant tumors, and it has a very high mortality rate^5^. As early-stage lung cancer has few specific symptoms, it is often diagnosed late, which results in a very serious public health burden^6^. Therefore, it is highly important to explore NSCLC pathogenesis and identify new diagnostic and therapeutic biomarkers^7,8^. Lung squamous cell carcinoma (LUSC) accounts for 40–51% of primary lung cancers^9^. LUSC treatment is less effective than that of lung adenocarcinoma, and there are fewer treatment options available for LUSC^10^. LUSC has unique epidemiological and clinicopathological features, such as a close association with smoking, a low EGFR mutation rate and a low ALK rearrangement rate, which leads to poor effects of targeted therapy for LUSC^11^.

COPD is often associated with LUSC, and they share common risk factors and pathogenesis^12^. The risk factors include a long history of smoking, age > 45 years, and high-risk occupations^13^. In terms of pathogenesis, a variety of mechanisms may combine to cause disease occurrence and development, including activated cell proliferation pathways, DNA repair gene dysfunction, a chronic inflammatory microenvironment, and an impaired immune response^14,15^.

**Figure 1:**
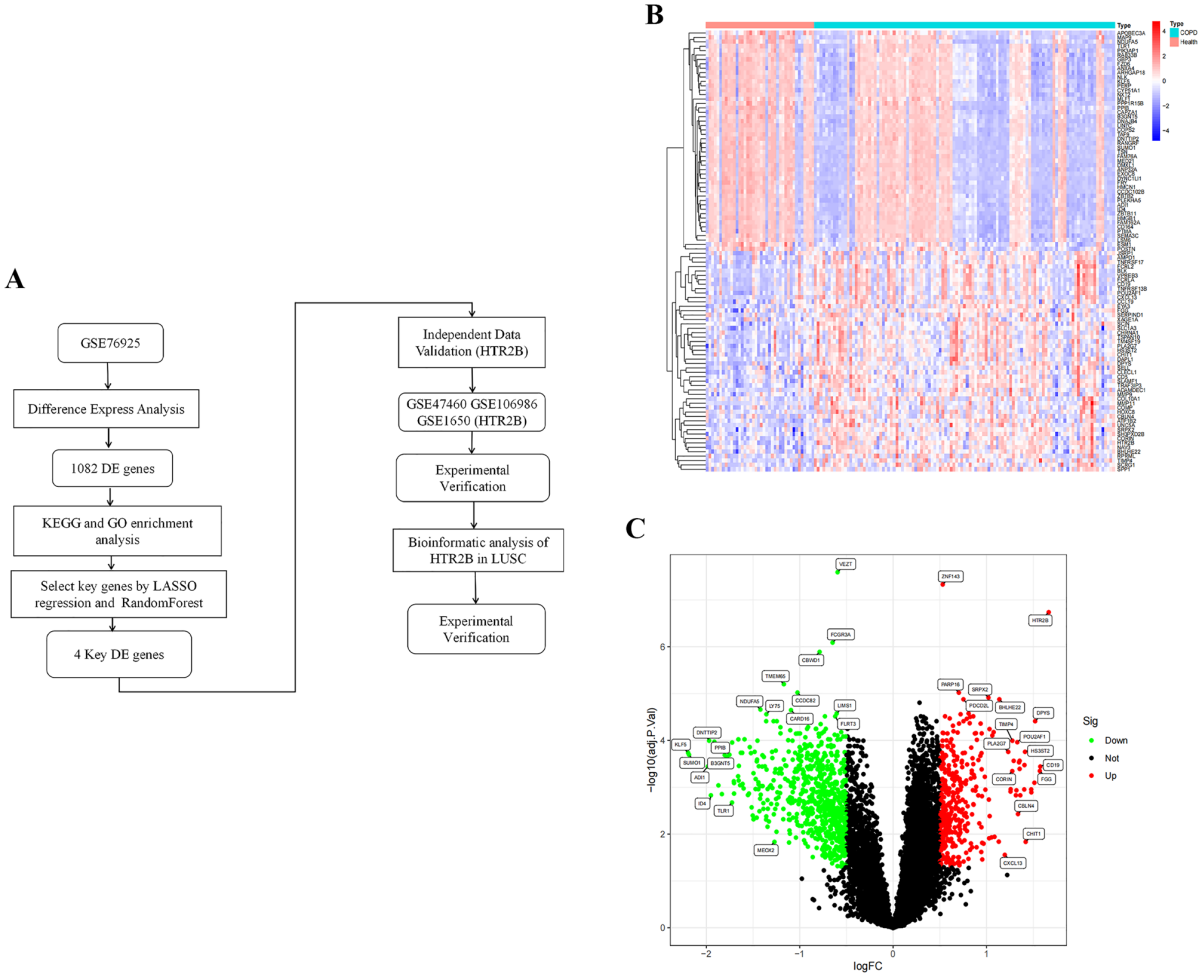
(**A**) Flowchart. (**B**) Heatmap of DEGs in the GSE76925 dataset. (**C**) Volcano plot of DEGs in the GSE76925 dataset.

In this study, we identified HTR2B as a novel key gene in COPD with LUSC through bioinformatics analysis and experimental validation. HTR2B is a promising therapeutic target and prognostic biomarker for COPD patients with LUSC (Fig. 1A).

## Methods

### Data acquisition and processing

The National Center for Biotechnology Information Gene Expression Omnibus (GEO) database (http://www.ncbi.nlm.nih.gov/geo) is an open-access database. In this study, we used the GSE76925, GSE47460, GSE106986, and GSE1650 GEO datasets. The GSE76925 dataset contained lung tissue data from 111 COPD patients and 40 healthy participants. The GSE47460 dataset contained data from lung homogenate samples from 75 COPD patients and 17 healthy participants. The GSE106986 dataset contained lung tissue data from 14 COPD patients and 5 healthy participants. The GSE1650 dataset contained lung tissue data from 18 patients with severe emphysema and 12 patients with mild or no emphysema. The Kaplan‒Meier plotter (http://kmplot.com/analysis/index.php?p=background) is a commonly used online database for differential gene expression and survival analyses. The database included expression data for all genes in > 30,000 samples from 21 tumors and complete patient clinical information. The Human Protein Atlas (HPA) (https://www.proteinatlas.org) provides 24,000 kinds of information on human protein distribution in different tissues and cells and displays immunohistochemical (IHC) staining results for more than 20 types of cancer. Information on HTR2B expression in LUSC patients and complete patient clinical data were downloaded from The Cancer Genome Atlas (TCGA) (https://portal.gdc.cancer.gov/) database.

### Screening for differentially expressed genes (DEGs)

We screened for DEGs between patients and controls in the GSE76925 dataset using the R limma package. *P* < 0.05 and a |log2 fold change (FC)|≥ 0.5 were set as the threshold values for DEG identification. The heatmap and the volcano plot of the DEGs were visualized with the “ggplot2” and “pheatmap” packages of RStudio software (3.1.3) (https://posit.co/download/rstudio-desktop/).

### Kyoto encyclopedia of genes and genomes (KEGG) and gene ontology (GO) enrichment analyses

KEGG pathway enrichment analysis was used to identify genes involved in multiple disease pathways and annotate gene functions (https://www.kegg.jp/kegg/kegg1.html)^16^. GO enrichment analysis was used to reveal the regulatory relationships of target genes related to the biological process, cellular component, and molecular function categories. The KEGG and GO enrichment analyses were conducted using the “org.Hs.eg.db” R package.

### Selection of candidate diagnostic markers

LASSO is a regression analytical arithmetic method that performs regularization to ameliorate forecast accuracy. LASSO regression analysis was conducted using the R glmnet package to investigate genes that were significantly associated with the discriminative power between COPD and healthy specimens. We then added the DEGs to the random forest classifier. We performed recurrent random forest classification for all possible numbers of variables and calculated the average error rate of the model.

### CIBERSORT analysis

The CIBERSORT (http://cibersort.stanford.edu/) computational approach involves a deconvolutional arithmetic based on gene expression and is used to assess the variations of a gene group relative to the remaining genes within a specimen. CIBERSORT was used to identify the immune cell responses and determine the associations between immune cells and the expression of critical genes in normal and COPD samples. Our primary objective was to identify the associations among these immune cells.

### Validation of the roles of key genes in the diagnosis of COPD

We used the “ggpubr” R package to construct receiver operating characteristic (ROC) curves for the key genes in the GSE76925, GSE47460, GSE106986, and GSE1650 datasets.

### Cell culture

BEAS-2B, H226, and H1703 cells were purchased from the Cell Bank of the Chinese Academy of Sciences (Shanghai, China). The cells were cultured in RPMI 1640 medium (Gibco, Carlsbad, CA, USA) supplemented with 10% fetal bovine serum (Gibco) at 37 °C with 5% CO_2_.

### COPD cell model construction

The COPD cell model was constructed using cigarette smoke extract (CSE; AAPR551-2, GuangZhou Peiyu Biotechnology Co., Ltd., Guangzhou, China). The experiment was conducted according to the manufacturer’s instructions.

### Cell transfection

Cells were transfected with a short interfering RNA against HTR2B (si-HTR2B, 5'-GCUGCAGUAUGCUACUAAUTT-3′) (GenePharma, Shanghai, China). Transfection was performed according to the jetPRIME instructions (PolyPlus Transfection, San Marcos, CA, USA).

### Construction of an HTR2B-overexpressing cell line

A fragment of the human HTR2B gene sequence was cloned into the pLVX-Puro vector. The BEAS-2B cell line was transfected with the control pLVX-Puro vector or with the pLVX-Puro-HTR2B plasmid (Cas9X, Soochow, China).

### Quantitative real-time PCR (RT‒qPCR)

Total RNA was extracted with the RNA-Easy isolation reagent (Vazyme, Nanjing, China) and reverse-transcribed into complementary DNA using a reverse transcription kit (Vazyme) according to the manufacturer’s protocol. RT‒qPCR was performed according to the RT‒qPCR reagent instructions (Vazyme). The primers used were synthesized by Tsingke (Beijing, China) (Table 1).

**Table 1:** RT‒qPCR primer sequences.

mRNA	Primer sequence (5′–3′)
HTR2B	Forward: 5′-TGGCTGATTTGCTGGTTGGAT-3′
	Reverse: 5′-TGAATGCTGTAGCCCGTGAGTT-3′
CDH2	Forward: 5′-GCCACCTACAAAGGCAGAAGAG-3′
	Reverse: 5′-CCTCAAATGAAACCGGGCTAT-3′
MMP2	Forward: 5′-AGTGGATGATGCCTTTGCTCG-3′
	Reverse: 5′-CAAGGTCCATAGCTCATCGTCAT-3′
ZEB1	Forward: 5′-CGCAGTCTGGGTGTAATCGTAA-3′
	Reverse: 5′-ATGTCTTGAGTCCTGTTCTTGGTC-3′
VIM	Forward: 5′-ATCTGGATTCACTCCCTCTGGTT-3′
	Reverse: 5′-CGTGATGCTGAGAAGTTTCGTTG-3′
SNAI1	Forward: 5′-CCTCAAGATGCACATCCGAAG-3′
	Reverse: 5′-AGCAGGGACATTCGGGAGA-3′
CCND1	Forward: 5′-AGAGGCGGAGGAGAACAAACAG-3′
	Reverse: 5′-GCGGTAGTAGGACAGGAAGTTGTT-3′
ITGAV	Forward: 5′-TGGGTTATTCTGTGGCTGTCG-3′
	Reverse: 5′-GCCATCTGCTCGCCAGTAAA-3′
ITGB1	Forward: 5′-GTTCAGTTTGCTGTGTGTTTGC-3′
	Reverse: 5′-ATCCTCTGGCTTGAGCTTCTCT-3′
GAPDH	Forward: 5′-GGAAGCTTGTCATCAATGGAAATC-3′
	Reverse: 5′-TGATGACCCTTTTGGCTCCC-3′

### Cell counting Kit-8 (CCK-8) assay

Cells were seeded in a 96-well plate (3.0 × 10^3^ cells per well), and each group had three replicate wells. The CCK-8 reagent (10 µL; Beyotime, Shanghai, China) was added to the respective wells at 24, 48, and 72 h. After 4 h of incubation, the absorbance of each well was measured at 450 nm in a microplate reader.

### Statistical analysis

The public gene expression data were analyzed with RStudio 3.1.3. Statistical analysis was conducted using GraphPad Prism 8.0. A *t* test was performed to compare data between two groups. Continuous variables are presented as the mean ± standard deviation. Differences were considered statistically significant at *p < 0.05, **p < 0.01, and ***p < 0.001.

## Results

### Identification of COPD-associated DEGs

We downloaded and analyzed the GSE76925 dataset from the GEO database. The dataset contained a total of 1082 DEGs, among which 371 were upregulated and 711 were downregulated. The heatmap and the volcano plot of the DEGs were shown in Fig. 1(B and C).

### KEGG and GO enrichment analyses

**Figure 2:**
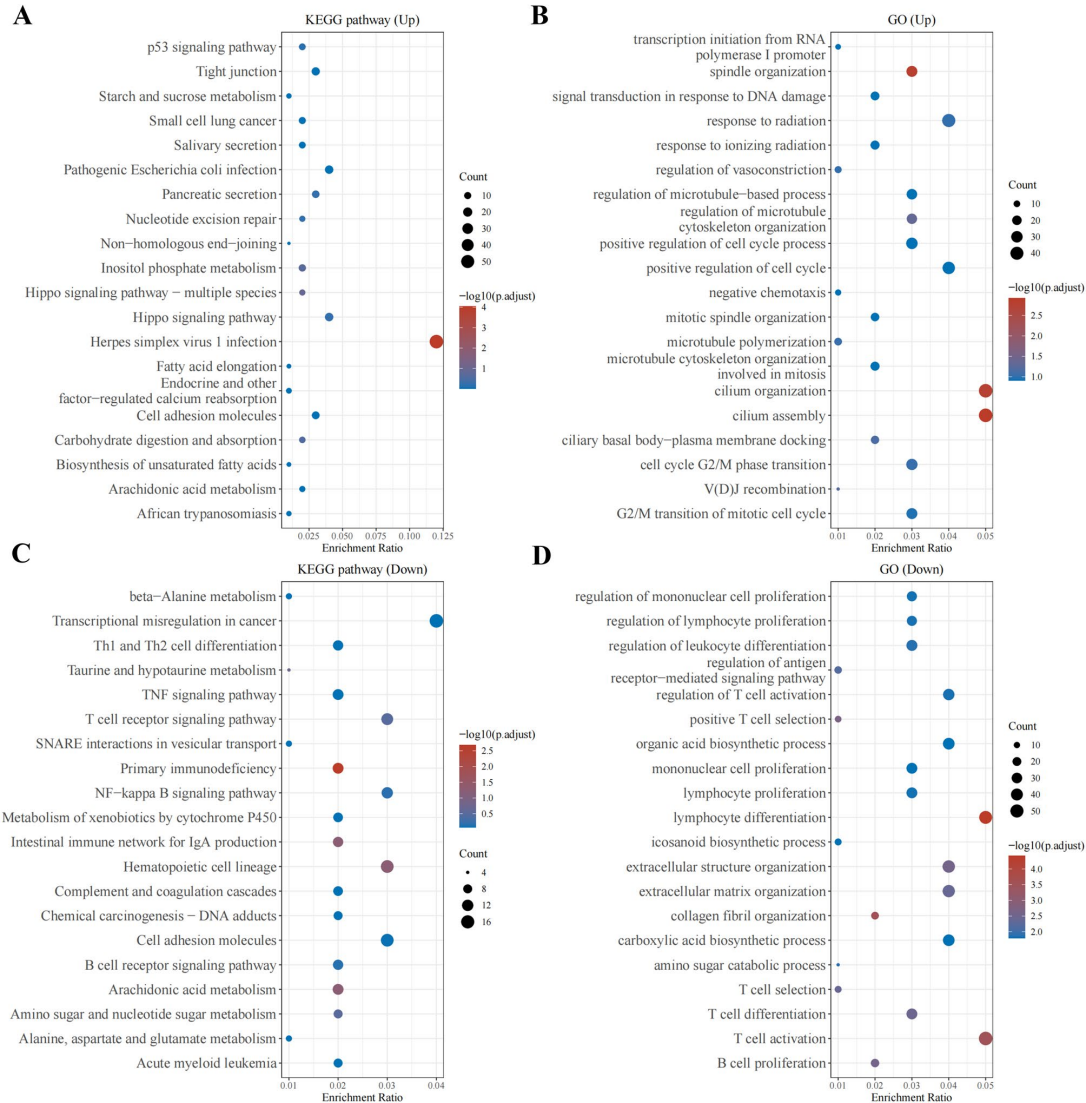
(**A**) KEGG pathway enrichment analysis results for the upregulated DEGs. (**B**) GO enrichment analysis results for the upregulated DEGs. (**C**) KEGG pathway enrichment analysis results for the downregulated DEGs. (**D**) GO enrichment analysis results for the downregulated DEGs.

KEGG analysis indicated that the DEGs were mainly enriched in the p53 signaling and β-alanine metabolism pathways (Fig. 2A, C). GO analysis indicated that the DEGs were mainly involved in transcription initiation from the RNA polymerase I promoter and in the regulation of mononuclear cell proliferation (Fig. 2B, D).

### Determination and verification of COPD markers

**Figure 3:**
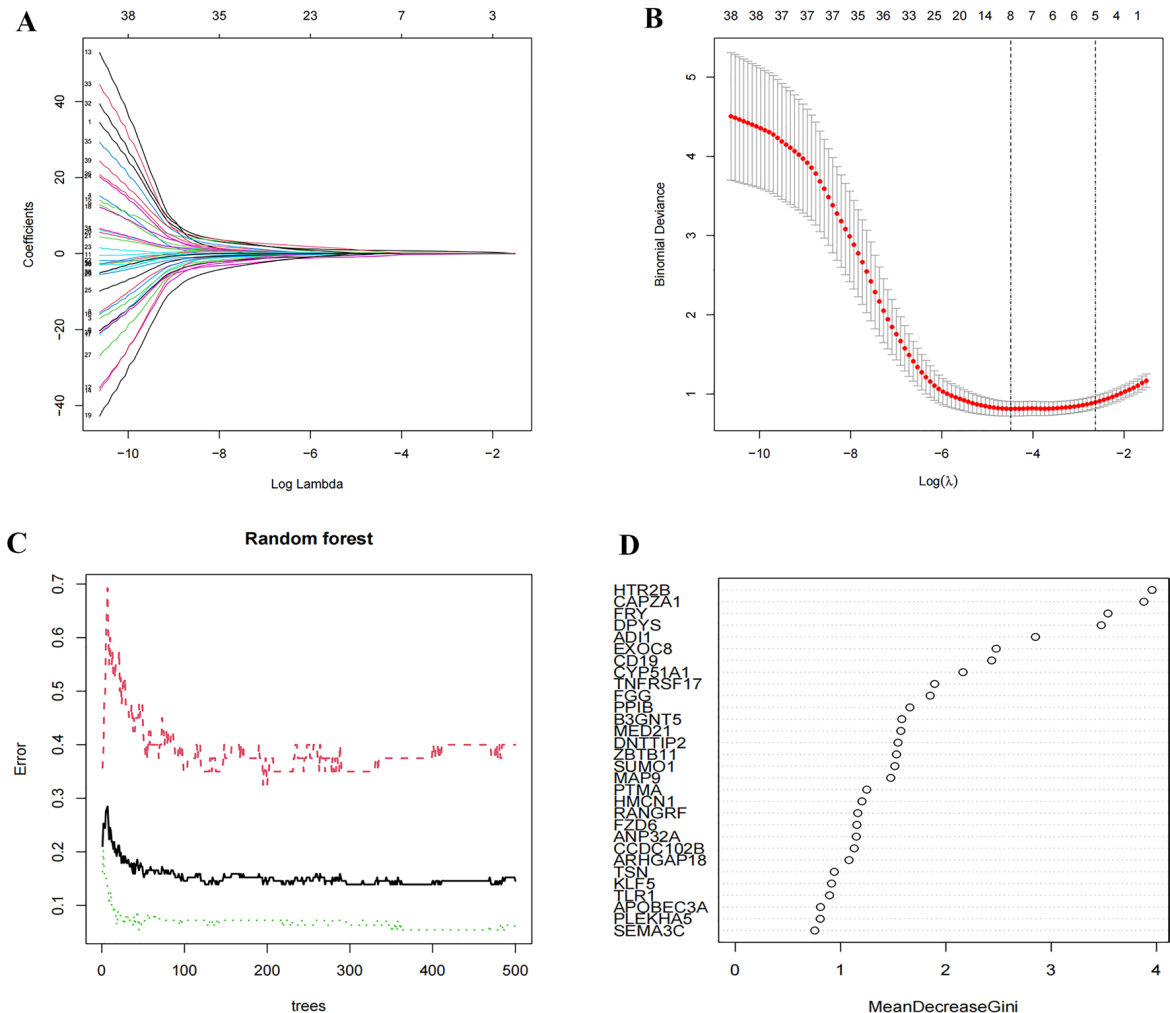
(**A**) LASSO coefficient profiles of the risk factors. (**B**) Eight risk factors selected by LASSO regression analysis. (**C**) Influence of the number of decision trees on the error rate. X-axis: Number of decision trees; Y-axis: Error rate. The error rate was relatively stable when the number of decision trees was approximately 400. (**D**) Results of the Gini coefficient method in the random forest classifier. X-axis: Genetic variables; Y-axis: Importance index.

The underlying biological markers of COPD were selected with two arithmetic algorithms. Each prognostic indicator coefficient track was analyzed using LASSO coefficient profiles by changing log (lambda) in the LASSO algorithm (Fig. 3A). The DEGs were identified by LASSO regression analysis, and eight variables were identified as COPD diagnostic markers (Fig. 3B). The number of variables was small, and the out-of-band error was low. Based on the relationship plot between the model error and the number of decision trees, 400 trees were selected as a parameter for the final model, which revealed a stable error in the model (Fig. 3C). Next, we identified eight DEGs with a significance value > 2 as candidate genes for subsequent analyses (Fig. 3D). Combining the results of the two algorithms yielded four key genes, namely, HTR2B, DPYS, FRY, and CD19.

### Key genes related to immunocyte infiltration

**Figure 4:**
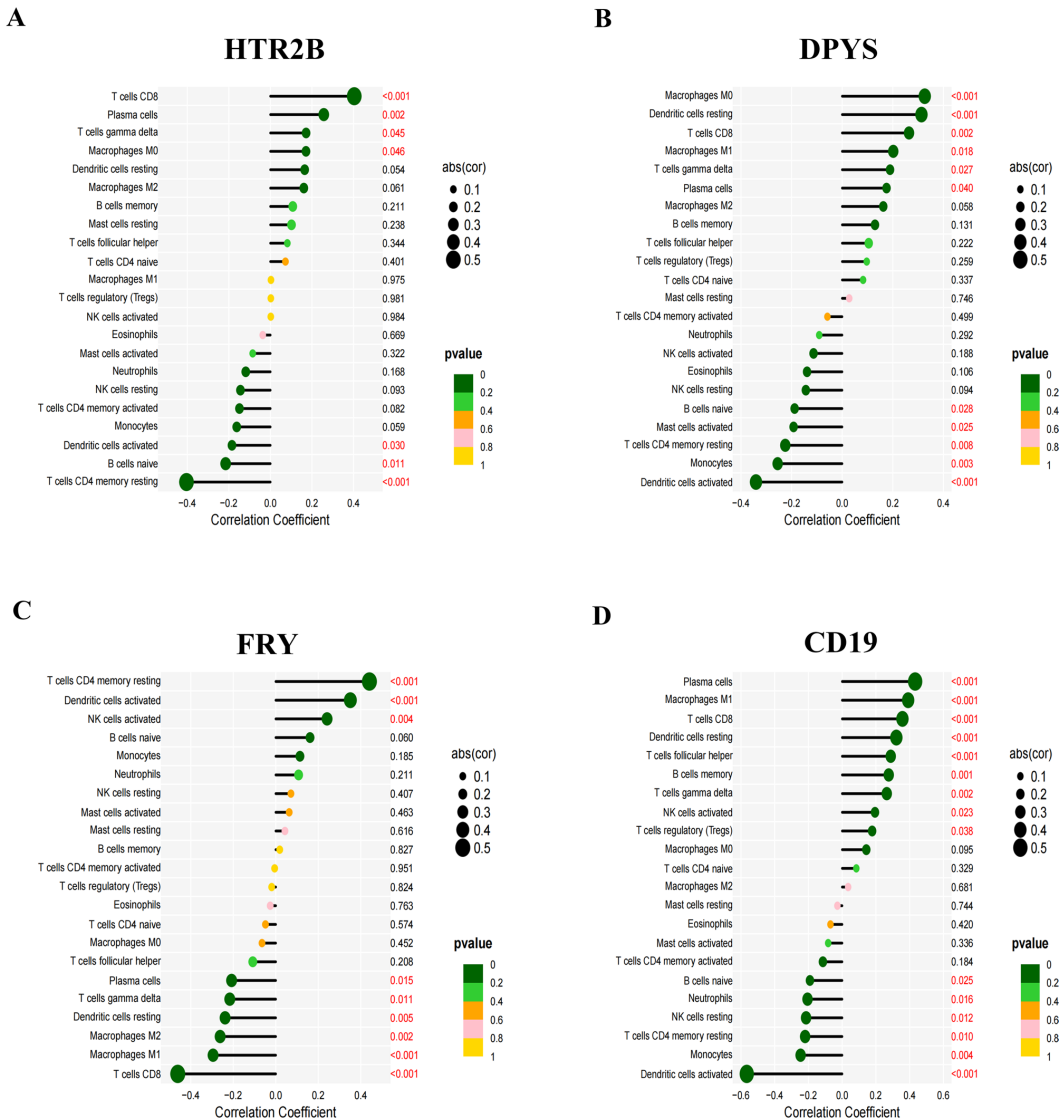
Correlations between key genes and infiltrating immune cells in COPD and normal samples.

We studied the coefficients of the four key genes (HTR2B, DPYS, FRY, and CD19) and the immunocyte infiltration status of COPD and normal samples to determine the correlation between immunocyte infiltration status and key gene expression. The immunocyte features were investigated with CIBERSORT, and the relationships between key gene expression and immunocyte infiltration levels were examined. We determined that HTR2B was correlated with CD8^+^ T cells (Fig. 4A), DPYS was correlated with M0 macrophages (Fig. 4B), FRY was correlated with resting memory CD4^+^ T cells (Fig. 4C), and CD19 was correlated with plasma cells (Fig. 4D). Our findings suggested that these genes might be involved in COPD progression by regulating the abovementioned immune cells.

### Validation of differential expression of HTR2B in COPD

**Figure 5:**
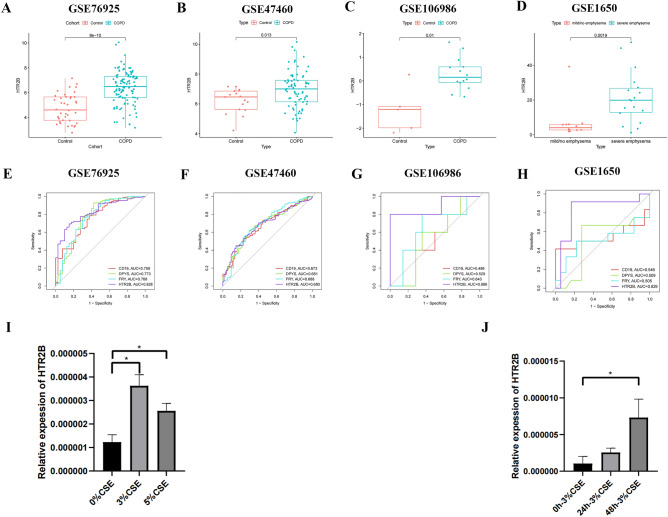
(**A**) HTR2B expression in the GSE76925 dataset. (**B**) HTR2B expression in the GSE47460 dataset. (**C**) HTR2B expression in the GSE106986 dataset. (**D**) HTR2B expression in the GSE1650 dataset. (**E**) ROC curves for key genes in the GSE76925 dataset. (**F**) ROC curves for key genes in the GSE47460 dataset. (**G**) ROC curves for key genes in the GSE106986 dataset. (**H**) ROC curves for key genes in the GSE1650 dataset. (**I**) HTR2B expression in CSE-stimulated BEAS-2B cells. (**J**) HTR2B expression in BEAS-2B cells stimulated with 3% CSE.

We analyzed the GSE76925, GSE47460, GSE106986, and GSE1650 datasets to verify HTR2B expression in COPD patients. Compared with that in the control group, HTR2B expression was significantly upregulated in the COPD group (GSE76925, GSE47460, and GSE106986) (Fig. 5A–C). HTR2B expression in severe emphysema patients was significantly greater than that in mild or no emphysema patients (GSE1650) (Fig. 5D). ROC curves for the key genes in the GSE76925, GSE47460, GSE106986, and GSE1650 datasets were plotted using the RStudio software (Fig. 5E–H).

### HTR2B was upregulated in the COPD cell model and promoted epithelial–mesenchymal transition (EMT)

**Figure 6:**
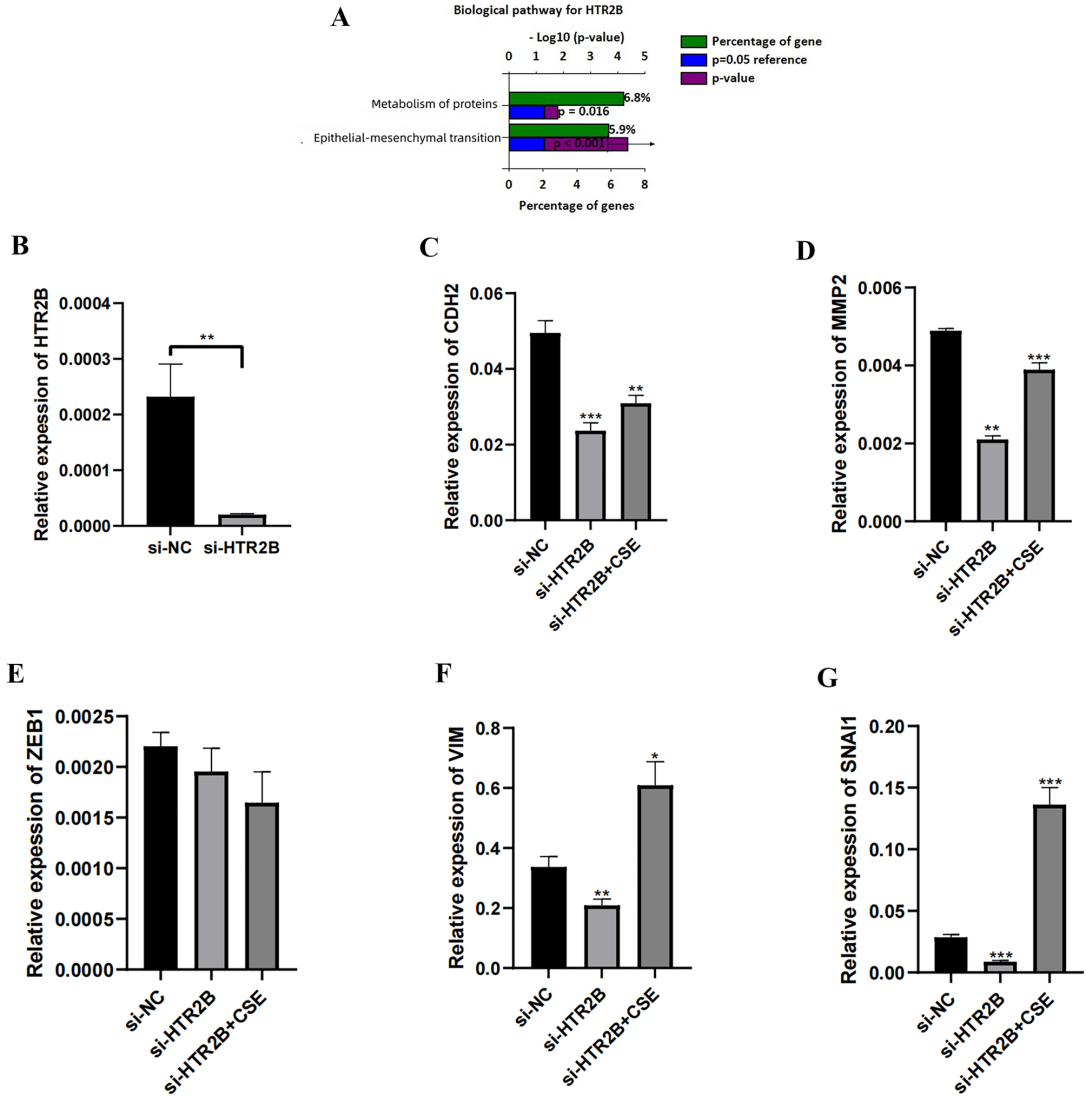
(**A**) Prediction of downstream signaling pathways regulated by HTR2B in COPD. (**B**) Inhibition of HTR2B expression in BEAS-2B cells. (**C–G**) Changes in the mRNA expression levels of EMT markers in BEAS-2B cells with different HTR2B expression levels.

According to the log2 FC (GSE76925), we selected HTR2B for preliminary experimental validation. We stimulated BEAS-2B cells with CSE to simulate the COPD microenvironment and determined that HTR2B expression was the highest after 48 h of stimulation with 3% CSE (Fig. 5I, J). Following GSE76925 dataset analysis, the genes coexpressed with HTR2B (cor > 0.3, *P* < 0.05) were input into FunRich 3.1.3 for signaling pathway analysis. The results demonstrated that HTR2B might be closely related to EMT (Fig. 6A).

We explored the molecular biological mechanisms of HTR2B in COPD by knocking down and overexpressing HTR2B in BEAS-2B cells (Fig. 6B and Supplementary Figure S1A). HTR2B knockdown led to significant downregulation of the EMT-related markers CDH2 (Fig. 6C), MMP2 (Fig. 6D), VIM (Fig. 6F), and SNAI1 (Fig. 6G), while ZEB1 expression did not significantly change (Fig. 6E). The addition of CSE to simulate a COPD cell microenvironment partially restored the CDH2 (Fig. 6C), MMP2 (Fig. 6D), VIM (Fig. 6F), and SNAI1 (Fig. 6G) expression levels by inhibiting HTR2B expression. HTR2B overexpression led to significant upregulation of CDH2 (Supplementary Figure S1B), MMP2 (Supplementary Figure S1C), ZEB1 (Supplementary Figure S1D), and SNAI1 (Supplementary Figure S1F) expression, while VIM expression (Supplementary Figure S1E) did not significantly change. CSE further upregulated CDH2 (Supplementary Figure S1B), MMP2 (Supplementary Figure S1C), and SNAI1 (Supplementary Figure S1F) expression via HTR2B overexpression.

### Bioinformatics analysis of HTR2B expression in LUSC

**Figure 7:**
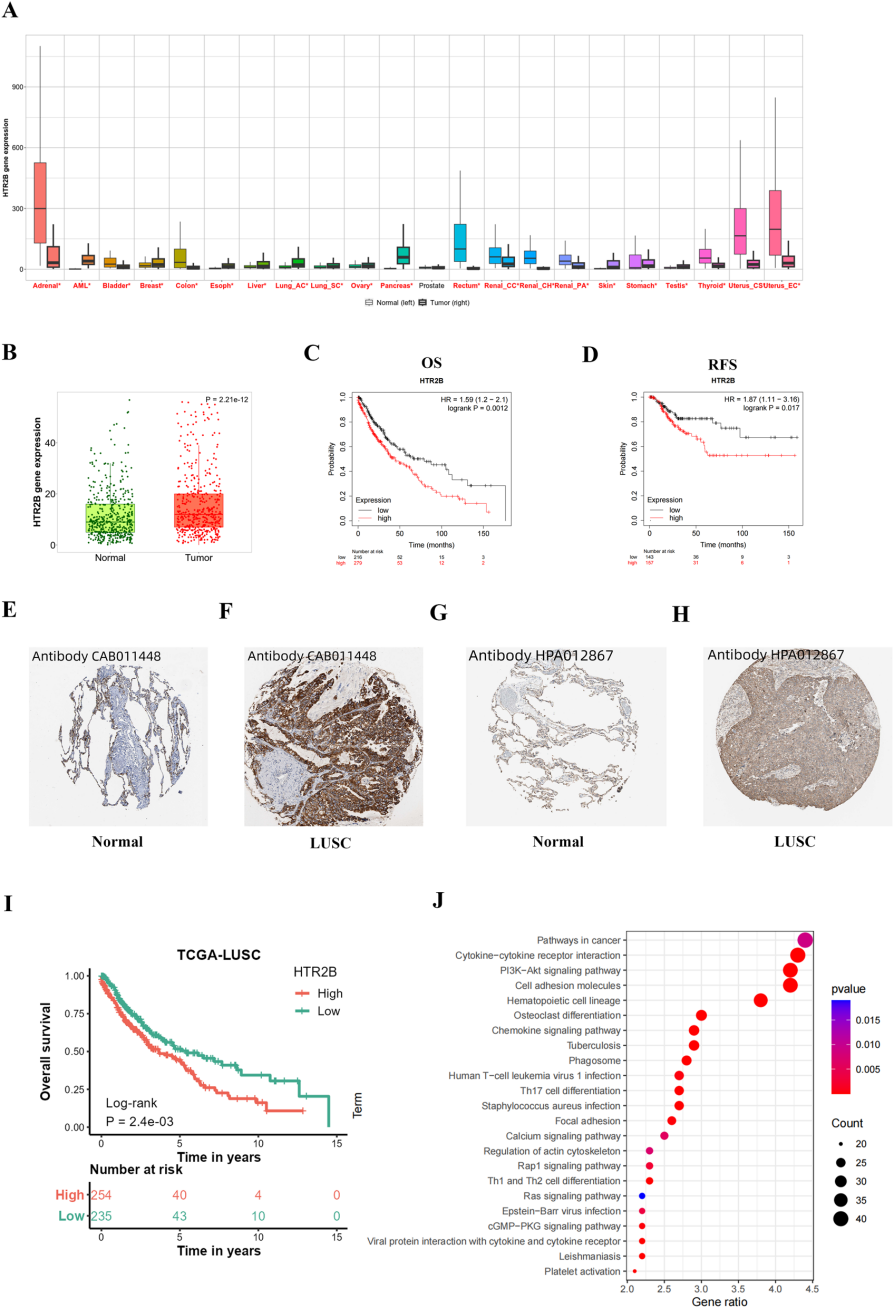
(**A, B**) Prediction of differences in the HTR2B mRNA expression levels between LUSC and normal tissue samples using the Kaplan‒Meier plotter database. (**C**) Results of survival analysis of LUSC patients stratified by HTR2B expression by using the Kaplan‒Meier plotter. (**D**) Results of RFS analysis of patients with LUSC stratified by HTR2B expression by using the Kaplan‒Meier plotter. (**E–H**) Results of IHC staining analysis of HTR2B in the HPA database. (**I**) Results of survival analysis of patients based on HTR2B expression in the TCGA database. (**J**) Prediction of downstream signaling pathways regulated by HTR2B in LUSC.

The Kaplan‒Meier plotter data indicated that HTR2B expression in LUSC tissue was significantly higher than that in normal lung tissue (Fig. 7A, B) and that lower HTR2B expression was linked to longer overall survival (OS) (Fig. 7C) and relapse-free survival (RFS) (Fig. 7D) in patients with LUSC. The IHC staining data in the HPA database further confirmed the upregulation of HTR2B at the protein level in LUSC (Fig. 7E–H). The TCGA LUSC data showed that a high expression level of HTR2B was significantly related to a poor patient prognosis (Fig. 7I). Following Kaplan‒Meier plotter database analysis, the genes coexpressed with HTR2B (cor > 0.3, *P* < 0.05) were input into FunRich 3.1.3 for signaling pathway analysis. The results demonstrated that HTR2B might be closely related to pathways in cancer, cytokine–cytokine receptor interaction, and the PI3K–Akt signaling pathway (Fig. 7J).

### HTR2B was upregulated in LUSC cells and promoted cell proliferation

**Figure 8:**
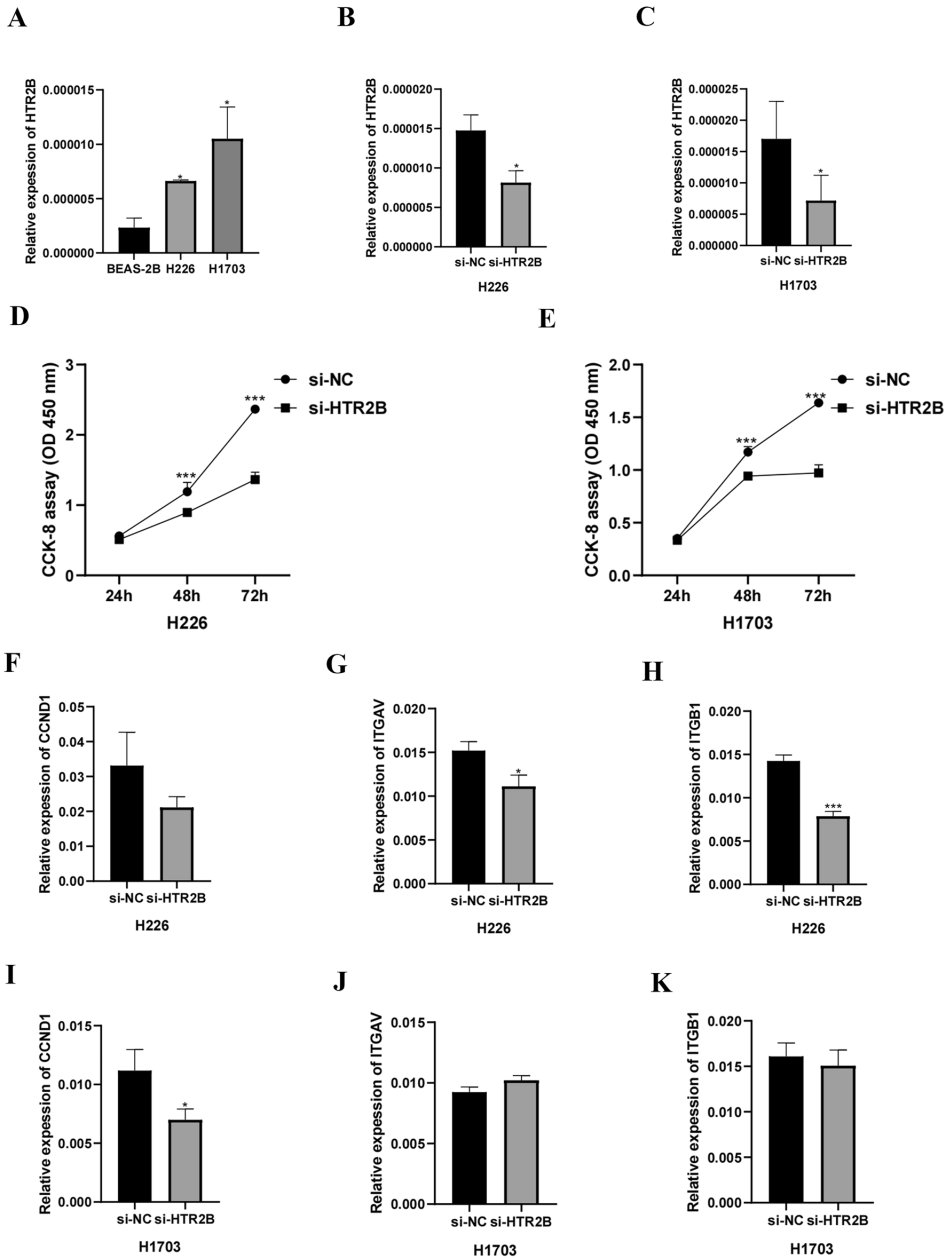
(**A**) HTR2B expression in BEAS-2B, H226, and H1703 cells. (**B, C**) Inhibition of HTR2B in H226 and H1703 cells. (**D, E**) LUSC cell proliferation, as determined by the CCK-8 assay. (**F, I**) CCND1 expression after short interfering RNA (siRNA)-mediated knockdown of HTR2B, as determined by RT‒qPCR. (**G, J**) ITGAV expression after siRNA-mediated knockdown of HTR2B, as determined by RT‒qPCR. (**H, K**) ITGB1 expression after siRNA-mediated knockdown of HTR2B, as determined by RT‒qPCR.

RT‒qPCR demonstrated that compared with that in BEAS-2B cells, HTR2B expression was significantly increased in H226 and H1703 cells (Fig. 8A). Furthermore, RT‒qPCR revealed that the HTR2B expression level significantly decreased following si-HTR2B transfection (Fig. 8B, C). The CCK-8 assay demonstrated that si-HTR2B transfection significantly reduced cell proliferation (Fig. 8D, E). In H226 cells, CCND1 expression did not significantly change following HTR2B knockdown (Fig. 8F), while ITGAV and ITGB1 expression significantly decreased (Fig. 8G, H). In H1703 cells, CCND1 expression significantly decreased following HTR2B knockdown (Fig. 8I), while ITGAV and ITGB1 expression did not significantly change (Fig. 8J, K).

### Pattern diagram

**Figure 9:**
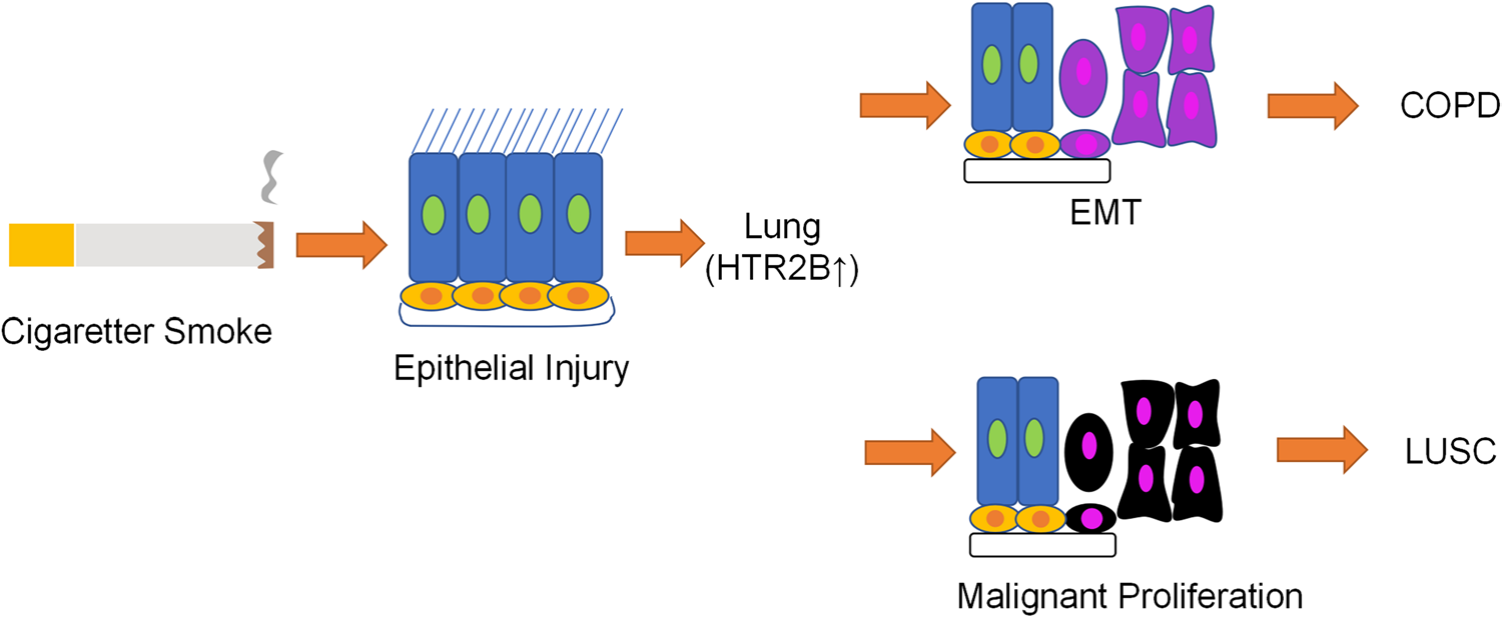
Pattern diagram.

The pattern diagram demonstrated that HTR2B might be a new biomarker and therapeutic target in COPD patients with LUSC (Fig. 9).

## Discussion

COPD is a respiratory disease, and its diagnosis currently depends mainly on lung function^17^. Researchers have concluded that existing spirometry-based diagnostic methods cannot sufficiently predict COPD progression and related deaths^18^. Presently, the role of genetic factors in COPD development is not well understood. Some genetic factors may increase COPD risk^19^. Many candidate genes, including those that encode α1-antitrypsin, secretory inclusion cell protease suppressor, and TNF-α, may increase COPD risk^20,21^. In the present study, we determined that HTR2B, DPYS, FRY, and CD19 might be promising biomarkers and potential therapeutic targets for COPD.

Lung cancer is the leading cause of cancer-related death worldwide^22^, and the 5-year mean survival rate is 20%, which decreases with the tumor stage^23^. This improvement can be attributed to advances in the diagnosis and treatment of patients with early and advanced lung cancer. Despite such advances in lung cancer treatment, increasing the efficiency of early detection is the most promising strategy for improving long-term survival of patients with lung cancer^24^. In recent years, the rapid development of high-throughput technology has generated large amounts of high-throughput sequencing data and expression profile data in oncology^25^. The combination of big data and different data types presents an entirely new perspective on the analysis of such data^26^. In the present study, we determined that HTR2B might be a diagnostic biomarker and potential therapeutic target in LUSC.

HTR2B belongs to the serotonin receptor family. HTR2B has been described as an oncogene in many cancers^27,28^. However, HTR2B has not been reported in lung cancer. Our results suggested that HTR2B might promote LUSC development. An animal study demonstrated that HTR2A and HTR2B partially mediated smoking-induced airway inflammation and small airway remodeling and that they might be new therapeutic targets for COPD airway remodeling^29^. Herein, we determined that HTR2B might contribute to COPD progression.

The chronic inflammatory response is considered to be one of the most important factors in the pathogenesis of COPD^30^. In COPD patients, respiratory tract inflammation can persist after smoking cessation, and there is persistent local infiltration of macrophages, neutrophils, and CD4^+^ and CD8^+^ T lymphocytes, which indicates the involvement of autoimmunity in COPD progression^31^. The main inflammatory reaction in COPD is an increase in the number of CD8^+^ T cells, while the number of CD4^+^ T cells is basically unchanged or slightly decreased, and the CD4^+^/CD8^+^ T-cell ratio decreases, which leads to the interaction between host T and B lymphocytes and results in an immune imbalance^32^. By analyzing the relationships between key genes and immune cell infiltration, we confirmed that the key genes might promote COPD progression by regulating immune cells.

During EMT, epithelial cells lose polarity and cell adhesion characteristics, gain the migration ability and aggressiveness, and become mesenchymal cells^33^. COPD can increase lung cancer risk by 40–50%, and 70% of lung cancers are accompanied by mild to moderate COPD, with EMT being one of the shared pathophysiological mechanisms^34^. Many studies have reported that the airways of COPD patients exhibit signs of increased EMT marker expression, reticulum basement membrane rupture, and decreased epithelial junction molecule expression, which suggests that EMT might promote small airway remodeling and fibrosis in COPD patients^35,36^. Our results confirmed that HTR2B might contribute to COPD progression by influencing EMT.

The occurrence and development of lung cancer involve the activation of various signal transduction pathways^37^. Studies on the PI3K/AKT signaling pathway have made remarkable progress in the field of cancer^38^. Our findings confirmed that HTR2B might promote LUSC cell proliferation by regulating the PI3K/AKT signaling pathway.

Cells are in constant contact with their surroundings, mainly through cell adhesion, and these contacts are essential for cell differentiation and growth^39^. Cell adhesion includes intercellular adhesion (direct contact between cells) and cell–matrix adhesion (the interaction between cells and the extracellular matrix)^40^. ITGAV and ITGB1 are downstream signaling genes of cell adhesion^41^. Our results confirmed that HTR2B might promote LUSC cell proliferation by regulating the cell adhesion signaling pathway.

Several limitations of the study should be noted. First, there is a lack of relevant in vivo experiments. Animal experiments are required for better clinical application in further studies. Second, the key genes need to be validated with a larger sample size. In the future, we plan to conduct clinical trials to verify the diagnostic efficacy of the key genes. Finally, the specific molecular mechanism by which HTR2B promotes the proliferation of LUSC cells is still unclear and needs to be explored via detailed molecular biology experiments.

In summary, we validated the differential expression and predictive ability of HTR2B in COPD patients across multiple datasets. We confirmed that HTR2B expression was upregulated in LUSC cell lines and that HTR2B knockdown significantly inhibited the proliferation of LUSC cells. Inhibiting HTR2B expression could inhibit the expression of EMT-related genes in COPD cell models and LUSC cells. HTR2B might be a new biomarker and therapeutic target in COPD patients with LUSC.

### Supplementary Information

Supplementary Information.

## Data Availability

All data involved in this study are available from the corresponding author upon request. The original GEO datasets used for analysis in this study (GSE76925, GSE47460, GSE106986, and GSE1650) are available from the GEO database (https://www.ncbi.nlm.nih.gov/geo/). Kaplan‒Meier plotter can be accessed at http://kmplot.com/analysis/index.php?p=background. The HPA can be accessed at https://www.proteinatlas.org. TCGA can be accessed at https://portal.gdc.cancer.gov/. The KEGG can be accessed at https://www.kegg.jp/kegg/kegg1.html.
