# Supplementary material for: HTR2B as a novel biomarker of chronic obstructive pulmonary disease with lung squamous cell carcinoma
Source: Sci Rep. 2024 Jun 8;14:13206. doi: 10.1038/s41598-024-63896-x (PMC11162446; doi:10.1038/s41598-024-63896-x)
Supplement: Supplementary file 1 — Supplementary Information. [file 41598_2024_63896_MOESM1_ESM.docx]

**HTR2B as a novel biomarker of chronic obstructive pulmonary disease with lung squamous cell carcinoma**

Yue Li^1#^, Yu Wang^1#^, Ruhao Wu^1^, Pengfei Li^1^*, Zhe Cheng^1^*

**Supplementary Figure S1**

**
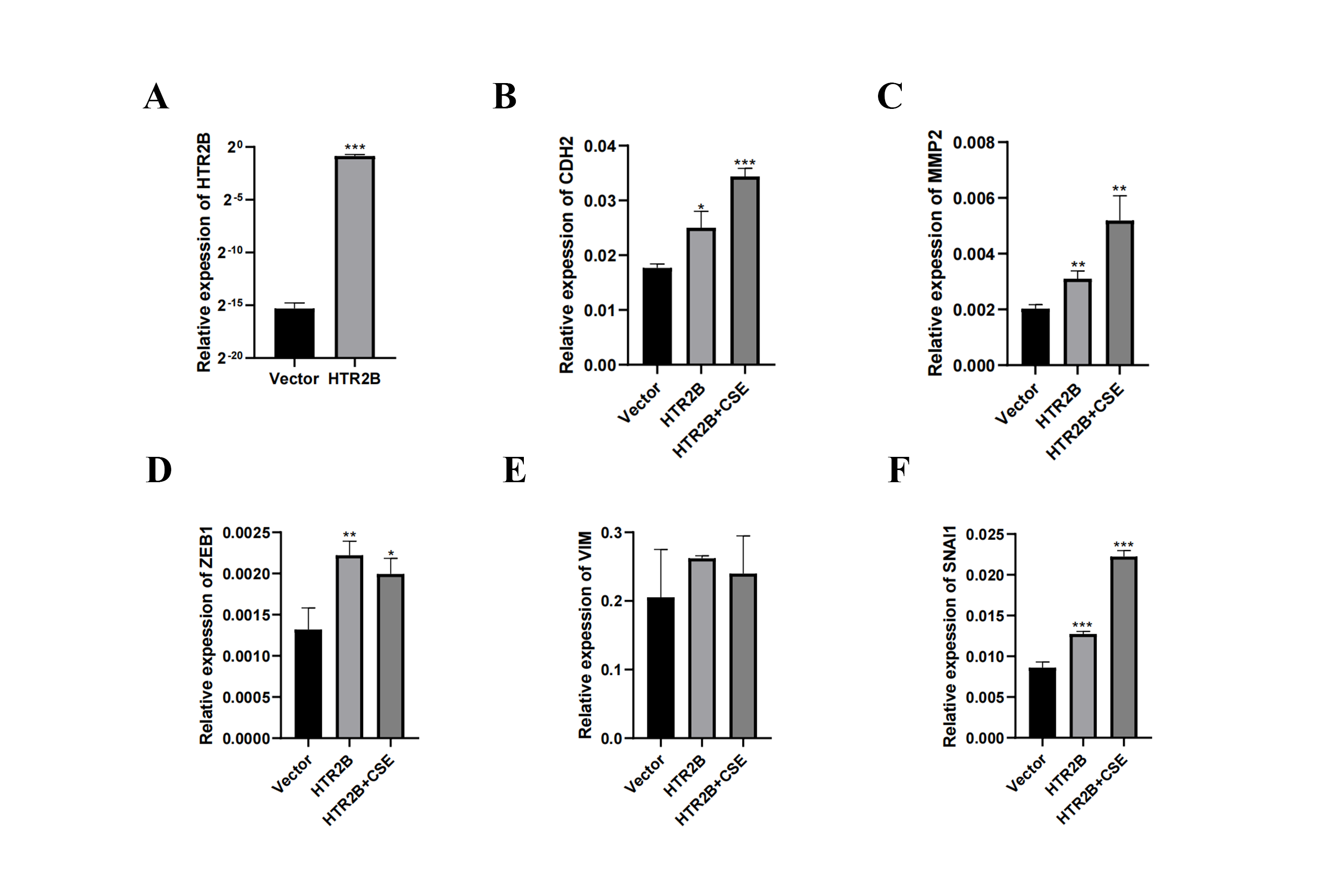
**

**(A)** *HTR2B* overexpression in BEAS-2B cells. **(B–F)** The changes in EMT marker mRNA expression levels in BEAS-2B cells with different *HTR2B* expression levels.
